# A Rare Case of Intraparenchymal Cerebrospinal Fluid Cyst Associated With Ventriculoperitoneal Shunt in an Adult Patient

**DOI:** 10.7759/cureus.17420

**Published:** 2021-08-24

**Authors:** Pinak Shah, Kartika Shetty, Maycky Tang, Elnaz Saberi, Nazanin Sheikhan

**Affiliations:** 1 Internal Medicine, Mountainview Hospital, Las Vegas, USA; 2 Internal Medicine, Riverside Community Hospital, Riverside, USA

**Keywords:** intraparenchymal cyst, vp shunt, csf, csf shunt, pericatheter csf cyst

## Abstract

Here we are reporting a rare phenomenon associated with ventriculoperitoneal (VP) shunt in the adult patient, namely, the development and finding of intraparenchymal pericatheter cerebrospinal fluid cyst. Our patient had a VP shunt placed for idiopathic intracranial hypertension 16 years ago before presentation to the hospital. The patient was admitted to the hospital for headache for past three weeks with the initial CT scan showing encephalomalacia and vasogenic edema. MRI showed the presence of a 4-cm intraparenchymal cyst in the right frontal lobe with surrounding vasogenic edema. The patient underwent two surgeries with the initial surgery for the drainage of cyst and second surgery for the placement of the cystoperitoneal shunt. Catheter-associated cysts are easily misdiagnosed due to their similarity in appearance to abscesses and other malignancies on imaging, and there are no guidelines yet on their evaluation and management. This is a unique case as the pericatheter cyst developed 16 years after the initial VP shunt placed. Given the rarity of this presentation, we hope that our case report can contribute to the development of guidelines and treatment options in adults with long-standing VP shunts.

## Introduction

An intraparenchymal pericatheter cyst is not a well-known complication of the ventriculoperitoneal (VP) shunt. Although it is rare, it is important for physicians to understand the unique radiological findings and treatment plan for it. Pseudotumor cerebri, otherwise known as idiopathic intracranial hypertension, is the presentation of elevated intracranial pressure without a space-occupying lesion. The primary treatment goal is to alleviate symptomology and to prevent vision loss through conservative treatments, such as weight loss and diuresis. When patients fail maximum medical therapy, surgical treatments, such as optic nerve sheath fenestration and cerebrospinal fluid (CSF) shunting, are considered. In our patient’s case, she presented with worsening headaches with magnetic resonance imaging demonstrating vasogenic edema and mass effect from a ring-enhancing cyst in the setting of a 16-year-old VP shunt placed for idiopathic intracranial hypertension.

## Case presentation

Our patient was a 54-year-old female with a history of pseudotumor cerebri, complicated by papilledema status following a right frontal VP shunt placement done 16 years ago, multiple sclerosis, and Wolff-Parkinson-White syndrome, who presented for neurosurgical evaluation due to the worsening persistent headache of three-week duration with CT imaging indicative of encephalomalacia with vasogenic edema. She reported progressive worsening throbbing and pulsatile headaches associated with right ear tinnitus and left vision changes, in the form of seeing rings of light and spots. She denied any nausea/vomiting, dizziness, focal deficits, or light or sound sensitivity, and further denied any loss of consciousness. Of note, two months prior, she was evaluated for right lower quadrant abdominal pain and found to have loculated fluid collection in the anterior wall of the right abdominal wall adjacent to the VP shunt, reportedly characteristic of shuntocele. Ultrasound-guided aspiration of the fluid at this time was sent for culture and sensitivity, which was ultimately negative for infection.

**Figure 1:**
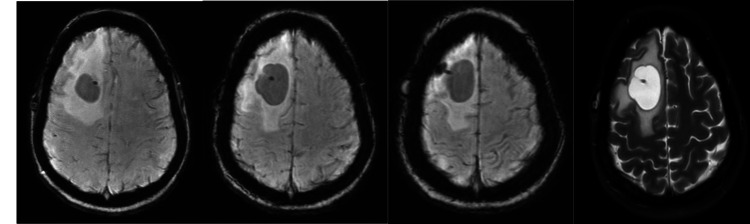
Initial brain MRI showing an approximately 4-cm intraparenchymal cyst in the right frontal lobe with surrounding vasogenic edema Minimal ring enhancement around the cyst is seen.

The initial brain MRI demonstrated an approximate 4-cm intraparenchymal cyst in the right frontal lobe with surrounding vasogenic edema, most characteristic of an intraparenchymal cyst related to the shunt (Figure 1). Due to features of ring enhancement of the cyst on initial imaging, empiric antibiotics were started.

The second brain MRI completed as part of the STEALTH presurgical mapping (the STEALTH System uses a patient’s MRI scans to maintain a continuous spatial relationship between the patient’s anatomy and the patient’s scan images during the surgical procedure using three-dimensional digitizers and powerful computer workstations) redemonstrated a 2.5 x 3.4 x 3.4 cm right frontal white matter cystic lesion with surrounding hypointensity suggesting vasogenic edema without definite abnormal enhancement, though mild sulcal effacement of the right frontal sulci was noted.

**Figure 2:**
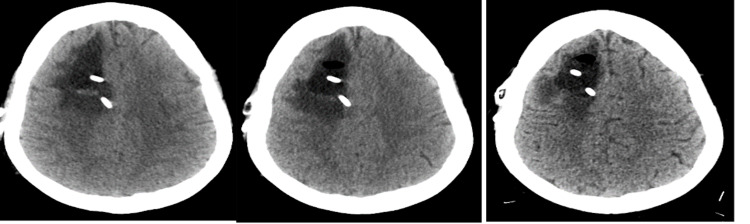
Post-operative CT scans of the head First two on the left are scans done on post-operative day 1 after cystoperitoneal shunt and show the placement of a second right frontal approach ventricular catheter and overall a slight decrease in size. Image on the right end is the CT scan done on post-operative day 4 after cystoperitoneal shunt and shows the unchanged appearance of the right frontal resection cavity with decreasing vasogenic edema.

The neurosurgery physician completed a burr hole aspiration of the right frontal cyst and aspiration of the existing VP shunt reservoir, and CSF fluids were sent for analysis. Due to the persistence of headache with nausea and vomiting despite continued acetazolamide use, she underwent a second surgery with cystoperitoneal shunt placement and revision of distal catheter of the existing VP shunt. Intraoperatively, distal portion of the existing VP shunt had no significant flow and after that the cystocatheter was placed and connected to the existing VP shunt via a Y-connector distal to the existing VP shunt valve. The immediate head CT done following cystoperitoneal shunt placement showed an overall slight interval decrease in the size of the cystic lesion (Figure 2).

A CT scan of the head done on post-operative day 4 demonstrated two ventricular catheters in place with an overall unchanged appearance of the right frontal resection cavity. Surrounding vasogenic edema was decreased. No significant mass effect or midline shift was observed (Figure 2).

After recovery from her surgery, she was discharged home with neurosurgery clinic follow-up instructions.

## Discussion

As of November 2018, there were 21 reported cases worldwide, 13 of which were in pediatric patients and eight in adults [1,2]. Even surprising is that only four of those adult cases presented after a shunt was placed during their adult years rather than placed when an infant or child. In most of the reported cases, shunt complications arise within several months or a few years following placement; rarely do the patients develop a complication many years following placement, much less, develop a pericatheter cyst. All of those four cases who had a VP shunt placed in their adult life developed cyst within one week and five years [3-6]. Our patient was unique in a way as she had her VP shunt placed in adulthood and had a complication of pericatheter cyst formation after 16 years. Shunt catheter obstruction is thought to be the most common cause of cyst formation; however, there are three reported cases in the literature where no obstruction was present [5,7]. Pericatheter cysts are more common among pediatric patients, probably because they develop more taut ventricles than adults after shunt placement, while their brain parenchyma is softer [8,9]. In the pediatric population, it would seem that the cyst can develop as soon as the shunt is placed, but may not become symptomatic until much later [1]. The fact that obstruction is probably the triggering factor of cyst formation leads to the conclusion that shunt revision is mandatory, even if the symptoms are not typical of shunt obstruction [6,10].

## Conclusions

Patients with idiopathic intracranial hypertension (also known as pseudotumor cerebri) who fail to respond to or are intolerant to medical therapy get treatment with CSF shunting and most commonly, a VP shunt. VP shunt is reserved for patients with idiopathic intracranial hypertension who develop worsening visual field defect or visual acuity loss due to papilledema. The most common complication of a VP shunt is shunt failure and other complications are infection, abdominal pain, overdrainage causing low pressure, cerebellar tonsillar herniation and syringomyelia, and intracranial hemorrhage. At this time, there are no current guidelines in place for regular monitoring and evaluation of our adult patients with VP shunts in place. It is unclear how often these adult patients receive screening imaging and how physicians can determine if any symptom flare-up is secondary to a shunt complication. CSF pericatheter cyst is a very rare complication of VP shunt placement. The malfunction of the peripheral tip of the catheter is considered to be the cause of cyst formation; thus, shunt revision and cyst drainage is the treatment of choice. With our case report, we hope to raise awareness of a rare complication of VP shunt that may occur even after many years of shunt placement. This case will help physicians to remain vigilant regarding this complication in patients with a VP shunt.
